# Agarose Stearate-Carbomer_940_ as Stabilizer and Rheology Modifier for Surfactant-Free Cosmetic Formulations

**DOI:** 10.3390/md19060344

**Published:** 2021-06-16

**Authors:** Qiong Xiao, Guo Chen, Yong-Hui Zhang, Fu-Quan Chen, Hui-Fen Weng, An-Feng Xiao

**Affiliations:** 1 College of Food and Biological Engineering, Jimei University, Xiamen 361021, China; xiaoqiong129@jmu.edu.cn (Q.X.); yhz@jmu.edu.cn (Y.-H.Z.); fqchenhy0109@jmu.edu.cn (F.-Q.C.); wenghuifen@jmu.edu.cn (H.-F.W.); 2 Department of Biotechnology and Bioengineering, Huaqiao University, Xiamen 361021, China; 3 National R&D Center for Red Alga Processing Technology, Xiamen 361021, China; 4 Fujian Provincial Engineering Technology Research Center of Marine Functional Food, Xiamen 361021, China

**Keywords:** agarose stearate, carbomer_940_, surfactant-free cosmetics, rheological behavior, sensory evaluation

## Abstract

Some commonly used surfactants in cosmetic products raise concerns due to their skin-irritating effects and environmental contamination. Multifunctional, high-performance polymers are good alternatives to overcome these problems. In this study, agarose stearate (AS) with emulsifying, thickening, and gel properties was synthesized. Surfactant-free cosmetic formulations were successfully prepared from AS and carbomer_940_ (CBM_940_) mixed systems. The correlation of rheological parameter with skin feeling was determined to study the usability of the mixed systems in cosmetics. Based on rheological analysis, the surfactant-free cosmetic cream (SFC) stabilized by AS-carbomer_940_ showed shear-thinning behavior and strongly synergistic action. The SFC exhibited a gel-like behavior and had rheological properties similar to commercial cosmetic creams. Scanning electron microscope images proved that the AS-CBM_940_ network played an important role in SFC’s stability. Oil content could reinforce the elastic characteristics of the AS-CBM_940_ matrix. The SFCs showed a good appearance and sensation during and after rubbing into skin. The knowledge gained from this study may be useful for designing surfactant-free cosmetic cream with rheological properties that can be tailored for particular commercial cosmetic applications. They may also be useful for producing medicine products with highly viscous or gel-like textures, such as some ointments and wound dressings.

## 1. Introduction

The cosmetic industry is one of the fastest growing industries and continues to develop through the absorption of new technologies and the integration of innovative sustainable products. According to Muda et al. [1], the global market for cosmetics, toiletries, and perfumes was about $382 billion in 2010.Cosmetics consist of a variety of formulas such as skin care, hair care, color cosmetics, and body care, while the most common type of cosmetics and personal care products are emulsions [2]. Since the emulsion is not a thermodynamically stable system, stabilizers are needed to prepare and stabilize the emulsion over time. In cosmetic emulsion, a unique feature of cosmetic formulations is that they consist of a mixed surfactant system rather than a single surfactant. Some of the most used surfactants in cosmetic emulsions include tween 80, span 80, sodium laureth sulfate (SLES), sodium lauryl sulfate (SLS), sodium dodecyl sulfate (SDS), cocamidopropylbetaine (CAPB), and polyethylene glycol ethers. Although the above surfactants in cosmetics are common, recent studies have shown that surfactants can cause adverse reactions such as skin irritation, hemolysis, and cytotoxicity [3]. In addition, surfactants are not biodegradable and have poor biocompatibility, which has a negative impact on the environment. Studies have shown that surfactants interact with the hydro-lipidic membranes of the skin, resulting in greater permeability, causing cell membrane disturbance, and sometimes resulting in cell lysis and protein denaturation [4]. As a result of increasing awareness and attention to these effects, a growing number of cosmetics have incorporated milder or more natural surfactants into their formulations, or partially replaced surfactants with polymeric thickeners, such as xanthan gum, cellulose derivatives (e.g., hydroxypropyl methyl cellulose), and microcrystalline cellulose. As for thickeners, they can also help improve stability and rheology, and even give better sensory properties to low-surfactant formulations [5].

Agarose (AG) is a natural polymer extracted from red seaweed. It is formed by binding β-d-galactose (G) and (1,4) α-3, 6-anhydrous galactose (A). This natural AG has many advantages, such as thickening, stability, gelling, and suspension. It is widely used in food, medicine, cosmetics, personal care, and other fields. However, in order to be an effective emulsifier, a natural polymer must have certain surface activity. A further practical consideration is that emulsifiers should not be strongly aggregated or gelled [6]. Obviously, the natural AG is not an effective emulsifying agent and cannot be a complete substitute for surfactants in the preparation of cosmetics due to its strong hydrophilic and gel properties. According to our previous study, hydrophobic chemical modifications could effectively reduce the gel strength of AG and give it amphiphilicity, and prepare ‘green’ AG-based surface active polymers having favorable emulsification properties [7]. The ability of AG to emulsify, thicken, and stabilize has been demonstrated in simple emulsion systems, and it is also important to determine its properties in complex substrates such as cosmetics. In addition, surfactant substitutes, such as AG, not only have to stabilize cosmetics, they also must replicate the relevant rheological and structural characteristics of conventional cosmetics. In practice, natural polymers are also not the only ingredients to improve the consistency and hardness of cosmetics, which need to be combined with synthetic polymers in order to adjust the properties during application [8]. For natural polymer-stabilized emulsions, plenty of researches have been done on the synergistic effects of these polymers on emulsion stability. For example, xanthan gum acts synergistically with guar gum, locust bean gum and konjacmannan, the result of this interaction is an increased viscosity or gel, and depends on the proportion of the gel in the mixture, pH value, and ionic environment [9]. Carbomer_940_ (CBM_940_), a class of cross-linked acrylic acid anionic polymers, builds viscosity, forms gels, stabilizes emulsions, and suspends particles in aqueous formulations, are nontoxic, and exhibit minimal or no irritation potential to skin and eyes at the concentrations employed in cosmetics and personal care products [10,11,12]. By comparing different polymers, we found that an agarose–carbomer_940_ physical mixture system exhibited good synergistic thickening properties and good swelling behavior with weak erosion. Therefore, we hypothesized that agarose stearate–carbomer_940_ (AS/CBM_940_) combination system might have the possibility of replacing, partially or completely, the surfactants in the cosmetic formulation, while maintaining a long-term stability and the characteristic rheological and organoleptic properties of the cosmetics.

To date, no studies focused on a rheological characterization of the AS/CBM_940_ systems and on the synergistic effect between AS and CBM_940_ in cosmetic formulations. The aim of the present study was first to synthesize the AS with emulsifying, thickening, and stabilizing capabilities. Mixtures of AS and CBM_940_ were then prepared and characterized to highlight the synergy between the two biopolymers, determine the specific ratio of the interaction, and characterize the effect of this synergy on rheological properties in commercial cosmetic ingredients. Then the AS-CBM_940_ ratio having the maximum synergistic influence on rheological properties was used in preparation of SFC with different oil content, and sensory characteristics of SFC were compared and analyzed with four different commercial moisturizing creams. The findings in this study will provide a practical guide to researchers to better design the surfactant-free cosmetics and advance their possible industrial applications.

## 2. Results and Discussion

### 2.1. Characterization of AS

**Figure 1:**
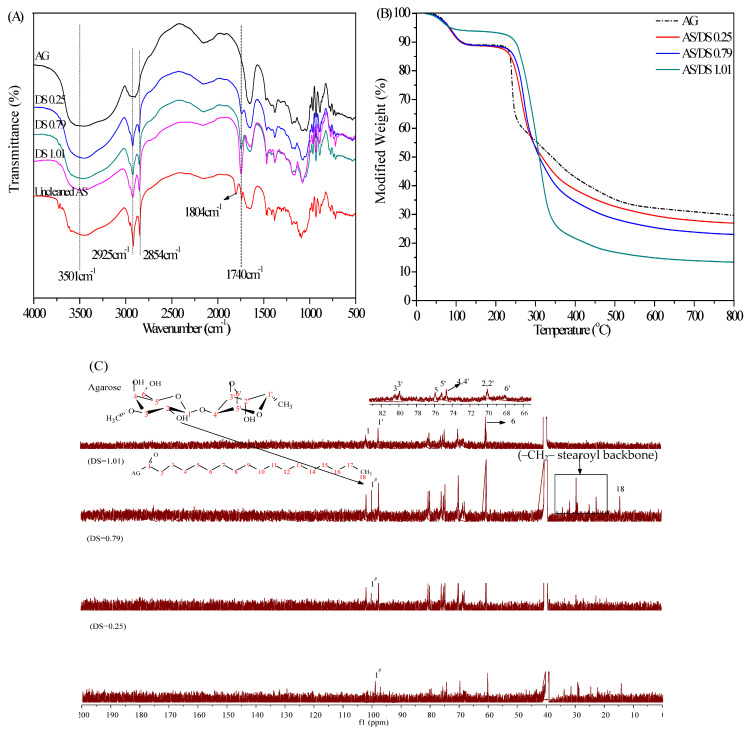
Characterization of AG and AS, (**A**) FT-IR spectra of AG, AS/DS 0.25, AS/DS 0.79, AS/DS1.01 and AS/uncleaned; (**B**) TGA curves of AG, AS/DS 0.25, AS/DS 0.79, AS/DS 1.01; (**C**) ^13^C-NMR of AG, AS/DS 0.25, AS/DS 0.79, AS/DS 1.01.

The FT-IR spectra of AS with different degree of substitution (DS) was shown in Figure 1A. The characteristic peaks representing the ester bond C=O appeared in all the three AS samples near 1740 cm^−1^, which proved the successful synthesis of AS and enhanced with the increase of DS. It also could be observed a significant increase in the intensity of the stretching signal of C−H that appears near 2925 cm^−1^ and a new band at 2854 cm^−1^, both corresponding to the stretching of the methyl and methylenes of the ester alkyl chain. In addition, AG showed a strong stretching vibration peak of –OH near 3500 cm^−1^, and the strength of this absorption peak decreased with the increase of the DS, indicating that the hydroxyl of AG was gradually replaced by the long-chain alkyl of acyl chloride. It could also be found that AS had an absorption peak at 1804 cm^−1^, which was mainly caused by the residual acyl chloride after the reaction. After cleaning, no other samples were found to have this characteristic peak here, indicating that the unreacted acyl chloride compounds could be removed after ethanol treatment. As shown in Figure 1B, TG results of AG and AS showed a two stage weight loss with the first minor one corresponding to loss of water around 50 °C to 125 °C with a weight loss of 11%. Once dehydrated, the sample was stable up to about 250 °C. With increase in temperature from 250 to 350 °C, the thermal decomposition took place again. The maximum degradation temperatures of AG and AS with different DS were 241 °C (AG), 273 °C (DS 0.25), 290 °C (DS 0.79), and 308 °C (DS 1.01), respectively. Thus, it was evident from TG data that AS was thermally more stable than AG and decomposition temperature increased with the increase in DS. Typical degradation mechanism for AS at higher temperatures is the decomposition of chemical bonds in the AG chain and oxidation. The improved thermal stability of AS with the increase of DS was mainly due to the low amount of remaining hydroxyl groups in the AG molecule after modification. That is, the more hydrophobic chains in the unit volume, the greater the steric resistance, which reduced the condensation capacity and improved the thermal stability of AS. In the earlier study, thermal properties of fatty acid starch esters also showed that the fewer the number of hydroxyl groups remained, the better was the thermal stability of the starch esters [13,14,15]. ^13^C-NMR spectra of AG and AS was shown in Figure 1C. After modified by acyl chloride, a new peak (C-1^#^) appeared at 99.85 ppm, indicating that the C-2 hydroxyl group was replaced by the alkyl chain, because the substitution of C-2 hydroxyl group affected the chemical displacement of the adjacent C-1. In addition, the C2–C6/C1′−C6′ peak of AG did not change after substitution, indicating that hydroxyl groups on C-4, C-6 and C-2′ were little or no substitutions, which was similar to the results of AG esterfied by anhydride [16].

### 2.2. Analysis of Emulsifying Properties

**Figure 2:**
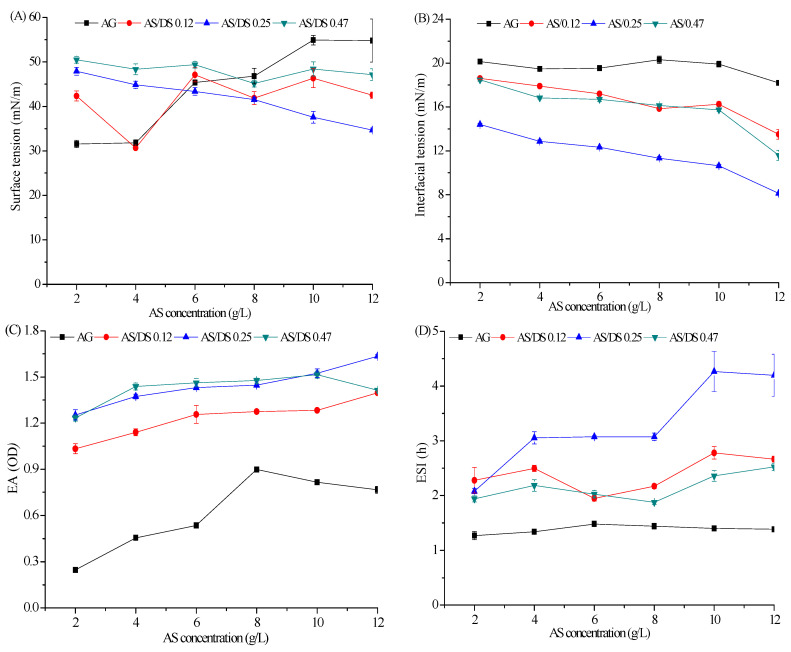
Emulsifying properties of AG and AS; (**A**) effect of AG and AS concentration on ST; (**B**) effect of AG and AS concentration on IT; (**C**) effect of AG and AS concentration on EA; (**D**) effect of AG and AS concentration on ESI.

Surface tension (ST) and interfacial tension (IT) are important indexes to evaluate the efficacy of emulsifiers. As shown in Figure 2A, The ST value of AG was reduced from 55.3 mN/m to 31.6 mN/m by AG at a concentration of 2 g/L, suggesting that AG is a surface-active compound at low concentration. However, with the increase of AG concentration, the ST gradually increased. This result was due to the gel formation of AG at >4 g/L concentration under the temperatures investigated, which affected their ST. The lowest ST value (30 mN/m) was observed for 4 g/L concentration of AS with DS 0.12. Similarly, with AS concentration increased from 2 g/L to 12 g/L, the IT of AS was decreased and the minimum interfacial tension (8.1 mN/m) was obtained when the concentration of AS (DS 0.25) rose to 12 g/L. In addition, the results showed that the emulsifying ability (EA) and emulsifying stability index (ESI) greatly improved after introducing hydrophobic group into the hydrophilic AG, and the EA increased with increasing concentration (Figure 2C,D). The results revealed that AS had the ability to reduce ST and IT and could therefore reduce the rate of emulsion creaming and improve the stability of the emulsion. On the whole, AS with a DS of 0.25 (Mw 131 kDa; gel strength 120 g/cm^2^ in 1.5 wt% gel) showed better emulsifying performance and could be used for further rheological experiments.

### 2.3. Influence of AS Concentration on the Rheological Properties of SFC

In general, the polymer concentration in emulsion has obvious influence on the microscopic structure and macroscopic behavior of samples. The viscoelasticity, thixotropy, and creep properties of SFCs through frequency sweeps, steady-shear flow, and creep–recovery tests were taken for evidence between rheology and microstructure.

#### 2.3.1. Steady-Shear Flow and Thixotropic Properties

**Figure 3:**
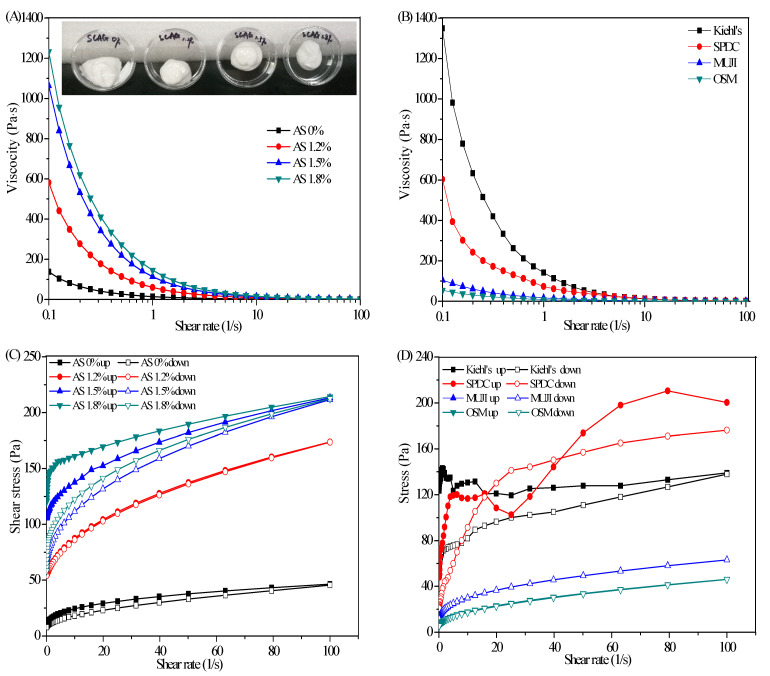

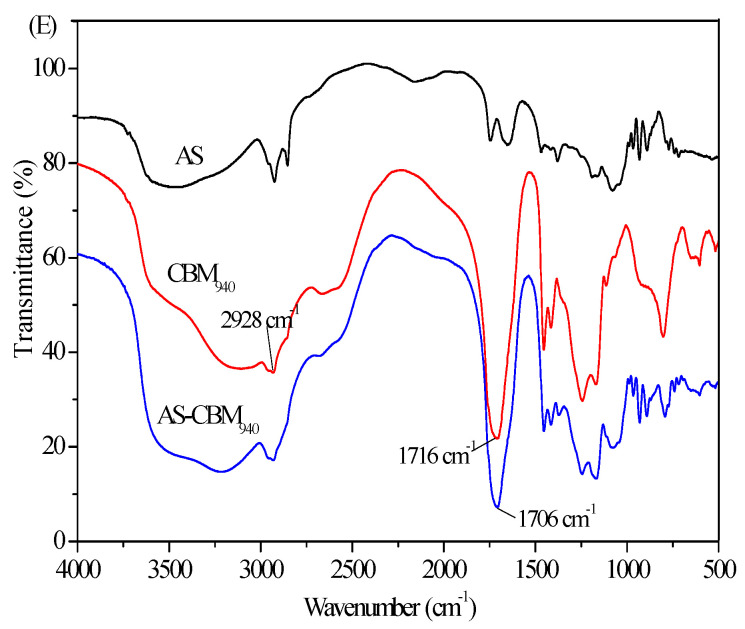
Viscosity curves and thixotropy curves for cosmetics and SFC and FTIR spectra of AS−CBM_940_. (**A**) Viscosity curves of SFC with different AS concentration; (**B**) viscosity curves of commercial moisturizers (Kiehl’s and SPDC) and lotions (MUJI and OSM); (**C**) thixotropy curves of emulsion gels with different AS concentration; (**D**) thixotropy curves of commercial moisturizers and lotions; (**E**) FTIR spectra of AS, CBM_940_, and AS−CBM_940_.

AS acted as an emulsifier, gelatinizer, and rheological regulator in SFC. Hence, studying AS concentration could guide in evaluating the rheological properties of SFC. As shown in Figure 3A, the viscosity of the SFCs continued to decrease with the increase in the shear rate, indicating a typical shear thinning behavior of non-Newtonian fluid, a typical feature of flocculating emulsion [17]. The shear thinning behavior was attributed to the disentanglement effect and orientation effect of the polymer chains [18,19]. In terms of the entanglement effect of molecular chains, in the mixed system of AS-CBM_940_, a series of physical junctions were formed by the entanglement or hydrogen bonding between the molecular chains of AS and CBM_940_; these physical junctions were constantly disintegrated and formed in the thermal motion of molecules, and their positions were also constantly changing, so they were vividly called ‘transient networks’. This ‘transient network’ was also affected by shear action. When the shear rate rose to a certain extent, the deformation rate of the physical junctions was greater than the rate of its re-formation, so the solution exhibited shear thinning behavior [20]. In terms of orientation effect, the AS-CBM_940_ molecules chains were randomly position in the absence of any external forces, while the polymer molecules chains would increasingly align in the direction of flow under the action of external shear force, which reduced interaction between adjacent polymer chains and result in less apparent viscosity [21].

Cosmetic creams and emulsions are generally applied on the skin. Owing to shear thinning, these substances are rapidly absorbed by the skin. As shown in Figure 3B, the maximum apparent viscosities of Kiehl’s and SPDC moisturizers were 1300 and 600 Pa·s, respectively, and those of MUJI and OSM emulsions were less than 100 Pa·s. The commercial moisturizing creams and emulsions both showed shear thinning behavior. Figure 1A showed that in the absence of AS, the viscosity of the sample prepared under the action of CBM_940_ was 150 Pa·s, which was close to that of the commercial moisturizing emulsions. In addition, the sample without added AS showed fluid-like characteristics and flowed to the bottom of the inverted plate, whereas those containing AS exhibited solid-like characteristics and remained at the middle of the inverted plates. When the AS concentration was 1.2 wt%, the sample viscosity was substantially higher than 600 Pa·s due to the synergetic effect between AS and CBM_940_. The synergetic effect could also be proved by the FTIR spectra of AS-CBM_940_. As shown in Figure 3E, there was ‘‘bathochromic shift’’phenomena for the –COOH vibration of CBM_940_ when combined with AS, i.e., the peak of CBM_940_ at 1716 cm^−1^ moved to the shorter wavenumber direction (1706 cm^−1^), therefore, we could infer that the hydrogen bonding between the –COOH of CBM_940_ and the –OH of the AS was confirmed as a key in the formation of gel [22]. Under this condition, the viscosity of the sample was close to that of the SPDC moisturizers, and the sample initially had a gel shape similar to that of a moisturizer. When the AS concentration reached 1.5 wt%, the sample viscosity increased remarkably, the viscosity was higher than 1100 Pa·s, and the gel morphology was stable. When the AS concentration was 1.8 wt%, the sample had lower viscosity than Kiehl’s moisturizer but was easily affected by the 3D polymeric network of AG, thus showing the characteristics of easy agglomeration and not delicate and consequently did not meet the requirements for a cosmetic cream.

Thixotropy, an important non-Newtonian fluid property, refers to a kind of time-varying ‘gel-sol’ interconversion behavior that occurs under the action of mechanical forces. The curve of thixotropic fluid is a closed loop between ‘upcurve’ and ‘downcurve’. The area enclosed by the up and down curve is referred to as hysteresis loop, which can be a preliminary indicator of thixotropic behavior [23]. In general, a large hysteresis loop indicates a strong thixotropic property [24]. For cosmetics, thixotropy affects the viscosity of products and the feeling after use. Products with thixotropy are easy to spread and be absorbed by the skin, which are important for cosmetics containing active substances. Figure 3C showed that when the polymer concentration was high, the area of thixotropic loop was large. Benchabaneet al. [18] suggested that the thixotropic behavior could also be attributed to the disentanglement effect and orientation effect of the polymer chains. Compared with commercial cosmetic emulsions, the CBM_940_ without AS had excellent thickening effect but had almost no thixotropy at low concentration. Therefore, the sample prepared under this condition showed low thixotropy, which was similar to that of commercial cosmetic emulsions. When the AS concentration increased to 1.2%, the thixotropy of the sample was also similar to that of the cosmetic emulsion (Figure 3D), indicating that the energy needed to destroy the structure of the sample was small, and the recovery ability of the structure was low. When the AS concentration increased to 1.5 wt% and 1.8 wt%, the thixotropy of the samples was strong and similar to that of commercial moisturizers. Therefore, the synergistic interaction between ASandCBM_940_ generated a reinforcing thixotropicity through additional hydrogen bond formation due to the carboxyl group participation of CBM_940_ and hydroxyl group participation of AS.

#### 2.3.2. Dynamic Viscoelastic Properties and Creep Recovery Test

**Figure 4:**
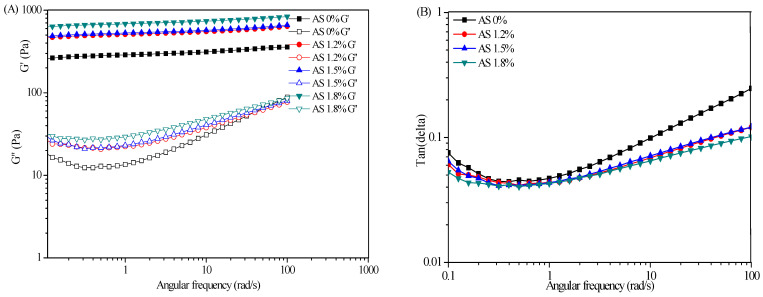

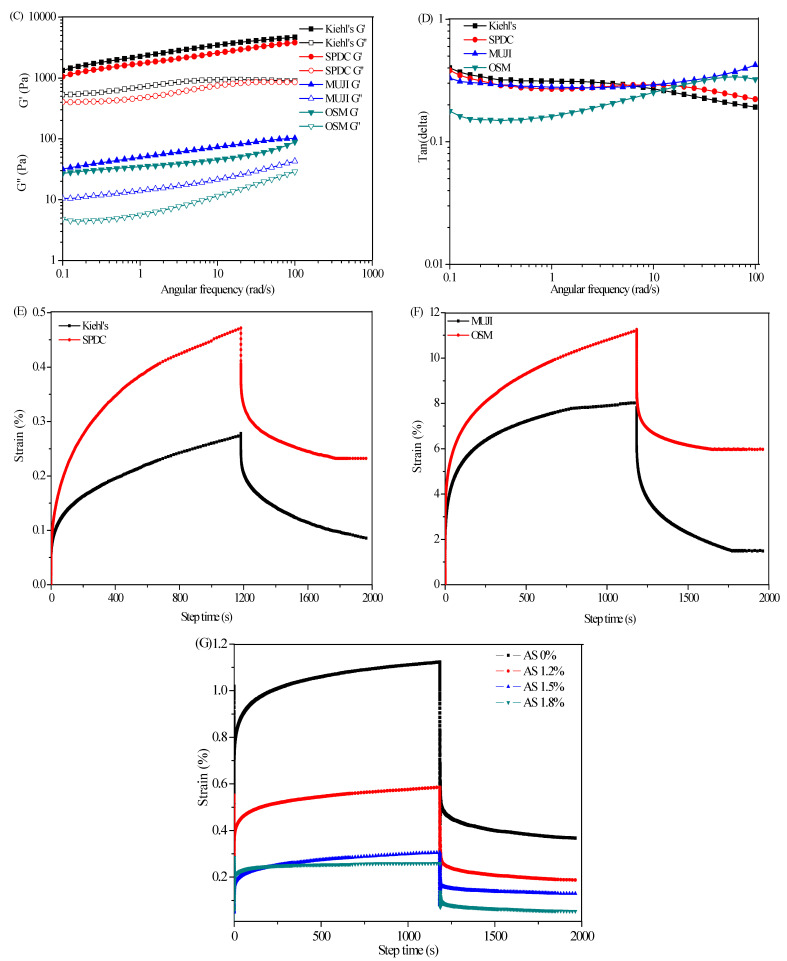
Dynamical oscillatory frequency sweep test curves and creep–recovery curves of SFCs with various AS concentrations and commercial moisturizers and lotions. (**A**) G′ and G″ of SFCs with various AS concentrations; (**B**) tanδ of SFCs with various AS concentrations; (**C**) G′ and G″ of commercial moisturizers and lotions; (**D**) tanδ of commercial moisturizers and lotions; (**E**) creep−recovery curves of the moisturizers; (**F**) creep–recovery curves of the lotions; (**G**) creep–recovery curves of the SFCs.

The dynamic viscoelastic properties of SFC at different AS concentrations from 1.2 wt% to 1.8 wt% were shown in Figure 4. The G′ was higher than G″ within the scope of the frequency tests (0.1–100 rad/s), and no cross point occurred, exhibiting a typical gel-like behaviors. When the AS concentration increased, the G′ in the linear region increased more than 2.5 fold from 244.43 Pa to 626.31 Pa for the SFC prepared at 0 wt% and 1.8 wt% AS, respectively, suggesting a strong effect of AS concentration on the gel strength of SFC. In addition, G′ increased slowly with the increase of frequency, indicating that the network structure formed by macromolecular association in the SFC was destroyed under the action of shear force. However, with the orientation of molecular chains, a new intermolecular association microregion was reconstituted, leading to the slow rise of G′ in the test range. Finally, both moduli tended to be close to each other, which could mean a similar microstructure existed at high frequency. Therefore, the SFC showed more as a solid rather than a liquid, that is, deformations were essentially elastic and recoverable [25].

These measurements also provide information on the loss factor tanδ (=G″/G′). Tanδ is used to evaluate whether an emulsion system exhibits a liquid- or solid-like behavior (purely elastic, δ = 0° and G′ > G′; purely viscous, δ = 90° and G″ > G′), that is, if the tanδ is large, then the viscosity ratio of the system is large and the fluidity is strong; otherwise, the elastic ratio is large. As shown in Figure 4B, the values of tanδ of the SFC were always less than 1 within the scope of the frequency tests (0.1–100 rad/s), illustrating the samples were dominated by their elastic properties rather than viscous properties even at low AS concentration. In addition, the G′ of cosmetic moisturizers and emulsions were greater than G″, and tanδ was also less than 1, showing a certain elastic rheological behavior (Figure 4C). Cosmetic moisturizers had higher G′ and thus higher elasticity than SFCs. In addition, the tanδ decreased with the increase in frequency, indicating that the system had a stronger ability to resist vibration than SFCs. In general, cosmetic cream or emulsion showed different G′ and G′ values due to the different preparation systems, but all showed characteristics similar to elastomer (G′ > G′).

Creep recovery tests can be used to study the transient behavior of the viscoelastic properties of a material [26]. According to creep recovery curve, a number of parameters can be extracted, but the following two parameters are the major indices that is commonly adopted to analyze the creep and revovery behaviour [27]. The maximum creep is expressed as the peak strain at the end of creep step. The relative recovery is expressed as the strain recovered at the end of recovery step divided by the peak strain. In general, a high peak strain is associated with a soft structure with high flow-ability, and a high relative recovery indicates a great elasticity and a ‘solid-like’ structure. As shown in Figure 4G, the peak strain for SFC without AS was four fold higher than that for emulsion prepared at 1.8%. On the contrary, the relative recovery for SFC with 1.5% AS was 42.3%, which was higher than the 31.9% and 20.7% for SFC prepared at 1.2% and 1.8%, respectively. Comparison of the curves of moisturizing creams and emulsions (Figure 4E,F) revealed that SPDC had the highest relative recovery (49.2%), and MUJI displayed the lowest relative recovery of 18.6%. In summary, polymer concentration affected the viscous and elastic components of the viscoelastic behavior of the SFC, and the SFC with 1.5 wt% AS concentration exhibited similar properties to cosmetic creams. 

### 2.4. Influence of CBM_940_ Concentration on Rheological Properties of SFC

**Figure 5:**
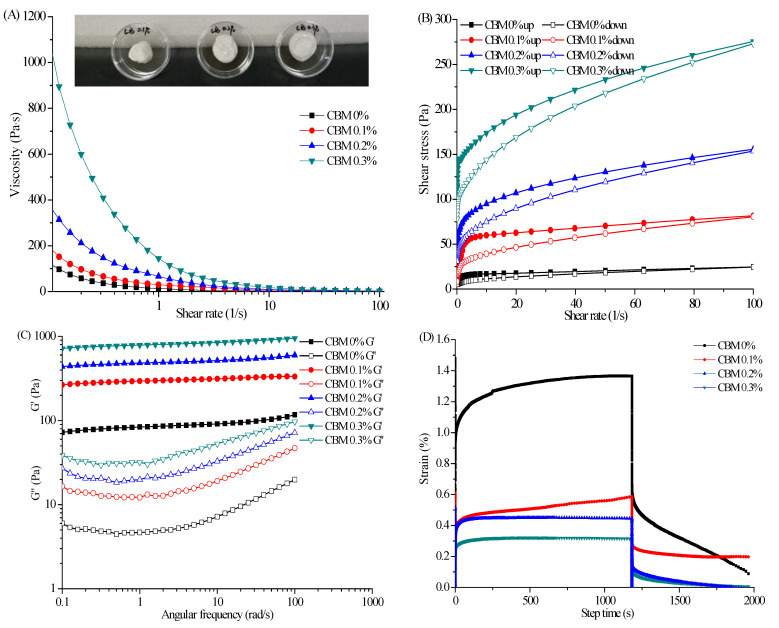
Flow behavior (**A**), thixotropy (**B**), dynamical viscoelasticity (**C**), and creep−recovery behaviors (**D**) of emulsion at different CBM_940_ concentrations.

The influence of CBM_940_ concentration (0 wt%, 0.1 wt%, 0.2 wt%, and 0.3 wt%) on the properties of SFC was evaluated. With the increase in CBM_940_ concentration, the viscosity increased substantially, and the maximum apparent viscosity occurred when the CBM_940_ concentration was 0.3 wt% (Figure 5A). The SFCs showed a typical shear-thinning behavior for all CBM_940_ concentrations. In general, CBM_940_ has a great thickening effect at a low concentration. The thickening mechanism of CBM_940_ can be divided into two types [27]: (1) neutralization thickening, that is, the CBM_940_ molecular chain is uncoiled under the water dispersion condition, after neutralization by alkali substances, i.e., sodium hydroxide and triethanolamine, the carboxyl group is ionized, and the coiled molecular chains are diffusely extended and greatly expanded due to the mutual repulsion of negative charges, thereby leading to microgel particle formation and thickening. When the concentration of microgel particles exceeds a certain value, the microgel particles are tightly and disorderly piled together and can remain stable when the external force is lower than a certain value. (2) Hydrogen bond thickening, that is, CBM_940_ molecule as a carboxyl donor can combine with one or more hydroxyl groups to form hydrogen bond and thicken. In this experiment, AS can be used as a hydroxyl donor to combine with carboxyl groups of CBM_940_ to form hydrogen bonds and thicken the solution. Therefore, at low concentrations, the binding rate of CBM_940_ and AS was low because the dispersion of CBM_940_ molecular chain was far from saturation, thus inducing a low viscosity. With the increase in CBM_940_ concentration, the molecular chain could be dispersed and stretched after CBM_940_ neutralized by alkali, and a large amount of CBM_940_ could also combine with AS hydroxyl group to thicken, thus showing a linear increase in viscosity.

Piau [28] found that a low concentration of CBM gel had excellent thickening effect but almost no thixotropy. Molleret al. [29] also confirmed that the thixotropic ring area of the CBM gel of 0.1 wt% was almost 0. As shown in Figure 5B, the thixotropic ring area was small under AS alone and without CBM_940_. In the absence of AS, the thixotropic ring area was still small when the CBM_940_ content was 0.3 wt%, indicating that the low concentration of CBM_940_ gel had low thixotropy (Figure 1B). However, with the increase in CBM_940_ concentration, the area of thixotropic ring increased gradually, indicating that the energy required to destroy the gel structure was increased with CBM_940_ concentration, and AS and CBM_940_ had a good viscosity synergism.

AS and CBM_940_ showed synergistic interactions that was strengthened at high proportions of CBM_940_. In this study, the CBM_940_ proportion showed great effect on the overall rheology of the SFC as confirmed by the curves from frequency sweeps. As shown in Figure 5C, the G′ of SFC gradually increased when the CBM_940_ level was raised from 0 wt% to 0.3 wt%, indicating that the gel strength increased with CBM_940_ concentration and showed the maximum at 0.3 wt% CBM_940_. In addition, G′ was larger than G″ within the scope of the frequency tests (0.1–100 rad/s), confirming the successful formation of emulsion–gels/SFC and the SFCs mainly showed as elastic behavior rather than viscous behavior. The image also indicated the formation of gelatinous SFCs at 0.1 wt%–0.3 wt% CBM_940_ (Figure 5A). As shown in Figure 5D, the peak strain for SFC prepared at 0.3 wt% CBM_940_ concentration was the lowest, and the highest was approximately 4.5 times higher than that of the lowest, suggesting that an increase in CBM_940_ concentration could increase the elastic component of the viscoelastic behavior.

### 2.5. Influence of pH on Rheological Properties

**Figure 6:**
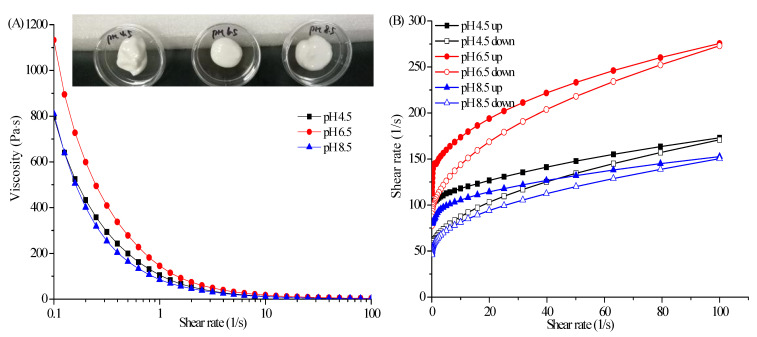

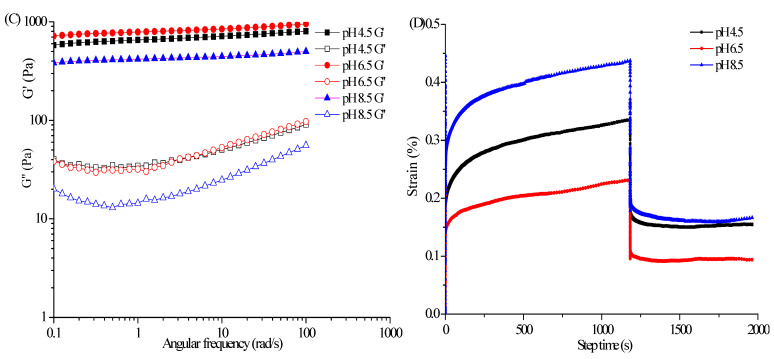
Flow behavior (**A**), thixotropy (**B**), dynamical viscoelasticity (**C**), and creep−recovery behaviors (**D**) of SFCs at different pH levels.

As shown in Figure 6A, the SFCs had a high apparent viscosity and exhibited a striking shear-thinning behavior at different pH values. The viscosity of SFCs highly depended on pH and the highest viscosity was obtained at pH 6.5 in a fixed shear rate. The viscosity changed with pH was attributed to the chemical structure of CBM_940_. CBM_940_ is a fully synthetic polyacrylic acid compound containing a large number of free carboxyl groups (56–68%). When CBM_940_ was dispersed in water, the molecular chain of carboxyl began to stray, leading to hydration and gradual molecular chain stretching. When the pH value was less than 4, the carboxyl group was seldom dissociated. When the pH value was greater than 4, the carboxyl began to dissociate due to the repulsive interaction of negative charges, and the CBM_940_ molecular chain spread out in a state of great expansion, thus increasing viscosity. The viscosity of CBM_940_ gel increased with pH and reached its maximum value in pH range of 6–8. When the pH continued to increase, the CBM_940_ gel tended to depolymerize. In addition, analysis of the emulsifying ability of AS revealed that when pH was greater than 6, the emulsifying ability of AS began to decline, especially under alkaline condition due the hydrolysis of agarose ester (data not show). The decrease in emulsifying ability enlarged the particle size of the emulsion. When the particle size in shear or collision is large, deformation and relative motion are easily to occur, and the viscosity of the system becomes smaller.

Oscillation frequency sweep curves indicated that G′ was obviously higher than G″ within the tested frequency range at each pH value, indicating that the SFCs mainly possessed elastic-like characteristics rather than viscous characteristic. Furthermore, the G′ of the SFC at pH 6.5 was higher than that of the SFCs at the other pH values. Although the viscosity of each SFC did not reach the ideal maximum value at pH 4.5 and pH 8.5; however, the overall associative cooperation of the polymer was still strong, and a complete 3D network was constructed. These findings were supported by visual observations of the samples, in which the SFCs at different pH showed solid-like characteristics and did not flow to the bottom of the inverted plate (Figure 6A).

As shown in Figure 6D, the peak strain for SFC prepared at pH 8.5 was higher than those for the SFCs prepared at other pH values, indicating that SFCs have a soft structure and high flow-ability. On the contrary, the relative recovery for SFC prepared at pH 6.5 was 45.9% compared with the 40.5% and 38.03% for SFCs prepared at pH 4.5 and pH 8.5, respectively. These findings suggested that the SFC had a great elasticity and a ‘solid-like’ structure. In summary, the rheological properties of SFC could be modulated by controlling their pH, which was mainly attributed to alterations in the interactions between the AS and CBM_940_.

### 2.6. Influence of Oil Content on Rheological Properties of SFC

**Figure 7:**
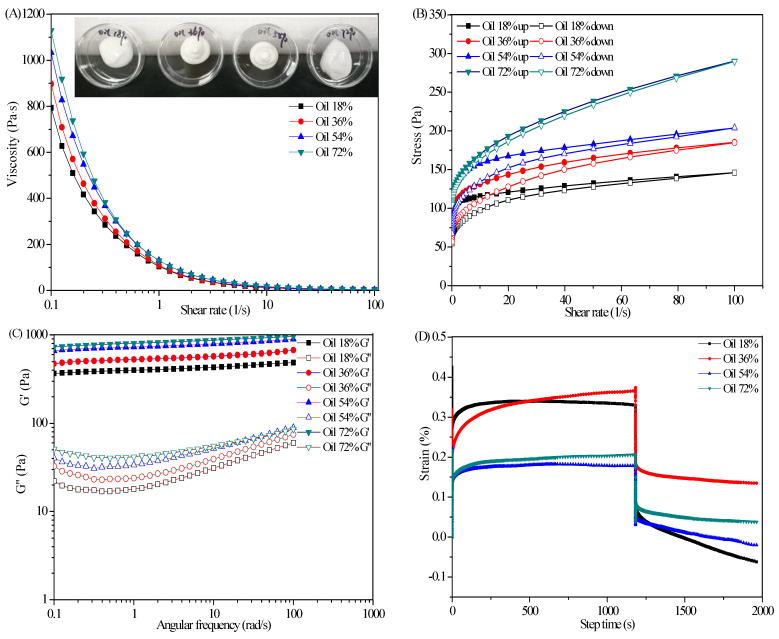

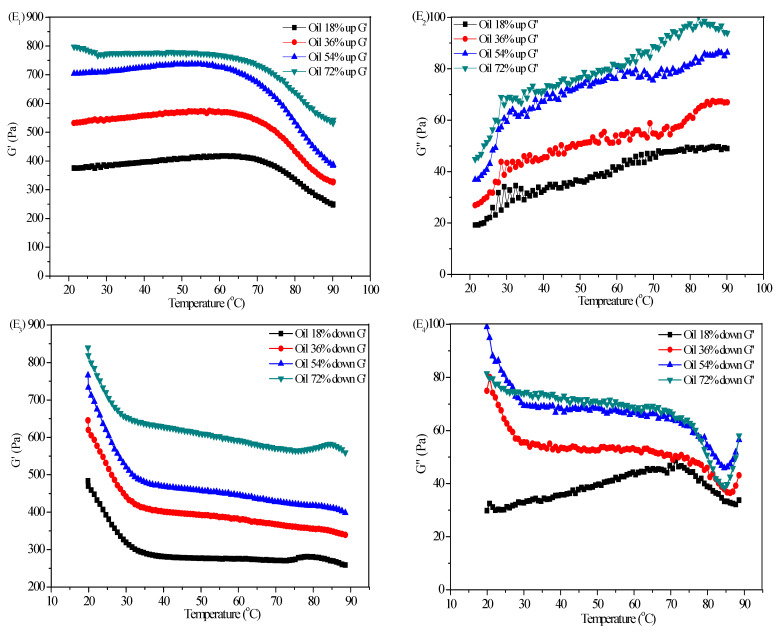
Flow behavior (**A**), thixotropy (**B**), dynamical viscoelasticity (**C**), creep–recovery behavior (**D**) and temperature ramp (**E1**): G′ curves of SFC for the gel melting process (up, 20−90 °C); (**E2**): G″ curves of SFC for the gel melting process; (**E3**): G′ curves of SFC for the gelation process (down, 90−20 °C); (**E4**): G″ curves of SFC for the gelation process).

The trend curves of the apparent viscosity of the SFCs with the shear rate at different oil content were tested under constant polymer contents (AS 1.5 wt%, CBM_940_ 0.3 wt%). As shown in Figure 7A, the apparent viscosity of the SFCs increased with the oil phase ratio at a fixed shear rate, but the apparent viscosity of the SFCs decreased with the increasing shear rate within the range of test shear rate, indicating a typical shear-thinning behavior. This difference in apparent viscosity was caused by the different interaction mechanisms of oil droplets in different oil phase emulsions. For the traditional emulsion–gel system, the droplets of dispersing phase could lose the fluidity of the system through compact accumulation under the condition of dispersing phase with high oil phase ratio. In general, the larger the volume of dispersing phase is, the more stable the emulsion gel system is. According to Manoiet al. [30], the increase of emulsion viscosity in high oil phase was mainly due to the flocculation network structure formed in the emulsion. For the emulsion–gel/SFC system in this research, the continuous phase was composed of the gel 3D network structure formed by AS and CBM_940_. The dispersing phase was similar to being filled in 3D network structure. Therefore, the SFC could be formed at a lowly dispersed phase volume, and the mechanical strength of the SFC depended on the strength of the continuous phase. However, the high viscosity under high oil phase condition was the result of the interaction between gel network of AS-CBM_940_ and flocculation network structure of oil phase.

Figure 7B showed that the thixotropic ring area of the SFCs first increased and then decreased with the increase in oil phase ratio, and the thixotropic ring area was the largest when the oil phase ratio was 54%. This finding indicated that the stability of the gel network could be maintained under this condition, and the accumulation degree of oil droplets in the network reached the maximum. When the oil phase ratio increased to 72%, the thixotropic ring area was the smallest, indicating that the inner network structure of the SFC was easily destroyed by shear action, that is, for the SFC formed through compact accumulation, the system lost its fluidity, thus requiring less energy to destroy its structure. In addition, its structure recovery ability was also weak. 

As shown in Figure 7C, the G′ of the SFCs with various oil phase ratios from 18% to 72% was larger than G″ within the range of test frequency. For example, G′ increased from 399.72 Pa (18%) to 801.59 Pa (72%) and G″ increased from 17.97 Pa (18%) to 41.61 Pa (72%) at a frequency of 1 rad/s, indicating that with the increase of oil phase ratio, the degree of compact accumulation between oil droplets increased, and oil droplets acted like active fillers in the gel structure. These result indicated that the strong networks in all emulsions was well-developed even for high oil phase ratio (72%, *v*/*v*). In addition, the phenomenon that G′ and G″ increased with increasing AS concentration (0 wt%–1.8 wt%) was observed for SFC with oil phase ratio of 36% (e.g., at 1 rad/s, G′ increased from 287.9 Pa to 684.2 Pa; G″ increased from 13.5 Pa to 29.2 Pa) (Figure 4A), but the increase was less than that for the SFCs with various oil phase ratios (18–72%) at the same polymer concentration (AS 1.5 wt%, CBM_940_ 0.3 wt%; at 1 rad/s, G′ increased from 399.72 Pa to 801.59 Pa; G″ increased from 17.97 Pa to 41.61 Pa). This phenomenon was attributed to the large contribution of oil droplets in the network structure at high oil phase ratio. The experiment results obtained was consistent with previous results that the oil droplets were considered to act as a ‘active fillers’ that interact with the gel matrix in the SFC network and further strengthened the gel network [31]. Therefore, the polymer concentration and oil content contributed to the gel network formation and determined the viscoelastic property of SFCs [32,33].

As shown in Figure 7D, the SFC with oil phase ratio of 54% showed the minimum peak strain, that with oil phase ratio of 36% had the maximum peak strain, and that with oil phase ratio of 54% showed the highest relative recovery 54.1% compared with the 45.1% and 36.0% for SFC with oil phase ratios of 72% and 36%, respectively. These findings suggested that the increase in the oil content contributed to an increase in the elastic component of the viscoelastic behavior to some extent. Dolz et al. [34] believed that under the action of constant stress, the spherical oil droplet became deformed, and the oil droplet surface area increased. The increase of droplet surface area leads to the increase of the deformation resistance of the emulsion system. Under constant stress, the drag on the oil bead increased the difficulty of deformation. Yilmaz et al. [35] and Rajinder [36] reported similar results, indicating that the strain of the sample decreased with the increase in oil drop fraction, and the deformation difficulty of the viscoelastic system increased.

AG is thermo-reversible, melts at high temperature, and sets back to gel on cooling. A representative temperature ramp curve for SFCs with different oil phase ratio was provided in Figure 7E. Figure 7(E_1_) showed that the G′ of SFCs in different oil phase ratio decreased with the increase in temperature mainly because the gel formed by AS melted with the increase in temperature. Hence, the force to maintain the structure of SFC was weakened. As shown in Figure 7(E_3_), when the temperature cooled from 90 °C to 20 °C, the G′ of the SFC was higher than that of the SFC before heating, indicating that the weak interaction force maintaining the SFC stable, such as hydrogen bond, hydrophobic interaction, and electrostatic repulsion, could be further rearranged and associated with each other after the temperature was reduced. Therefore, the strength of the SFC was enhanced. Comparison of the G′ and G″ of SFCs in the whole test range revealed that the G′ of SFC with different oil phase ratio was always higher than G″ an order of magnitude, that is, no crossover point was seen in the graph corresponding to the melt in G’ of gel (gel-to-sol transition) during the heating cycle and ‘re-gelation’ (sol-to-gel transition) during cooling. Hence, the thermal stability of SFC was confirmed.

### 2.7. Morphology and Structureof the SFC

**Figure 8:**
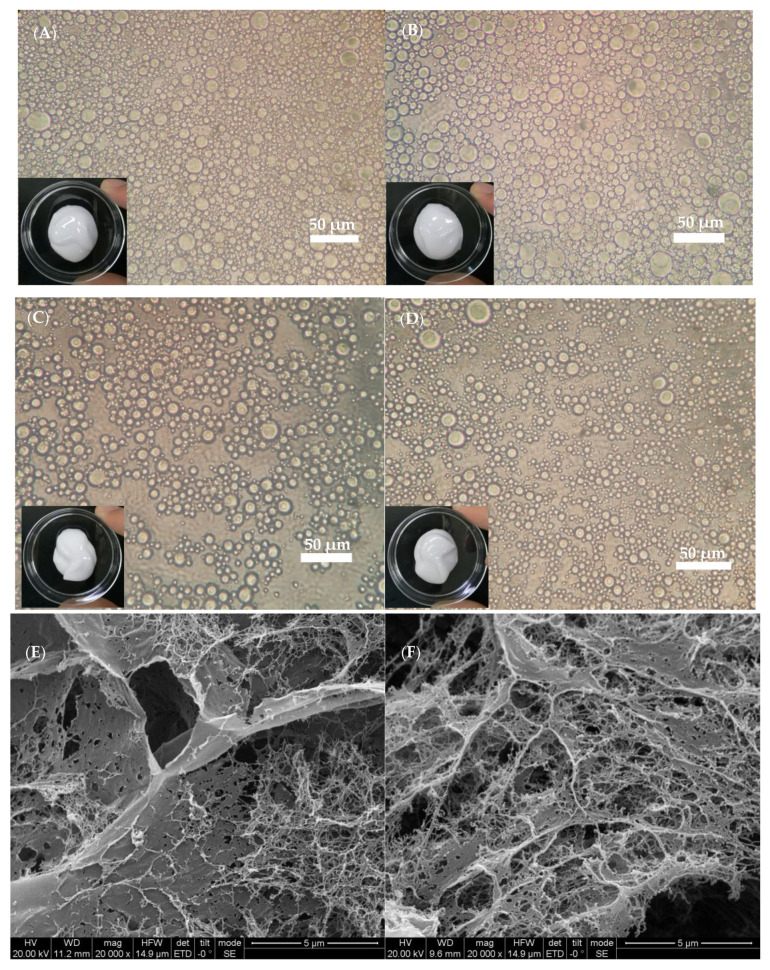
Optical microscopy of SFC and SEM micrographs of the SFC network after deoiling and freeze-dried. (**A**) Oil volume fraction of 72%; (**B**) oil volume fraction of 54%; (**C**) oil volume fraction of 36%; (**D**) oil volume fraction of 18%; (**E**) oil volume fraction of 18%; (**F**) oil volume fraction of 72%.

As shown in Figure 8, the microtopography of the SFC was similar to a concentrated emulsion with evenly distributed oil droplets. The density of the emulsion droplets gradually increased, which was consistent with the high viscosity and elastic modulus data. Under high oil phase ratio conditions, the strong interaction between oil droplets could still be observed, indicating that the formed emulsion had a high mechanical strength value. Different microtopography indicated that the formation of SFC structure had different mechanisms. The microstructures of SFCs at various oil contents (18% and 72%) were characterized using SEM to understand their structure formation. As shown in Figure 8E,F, the network structure was dominated by the polymer network with empty spaces (or holes). Differences in the network were attributed to the ratio of the polymers at the oil-water interface and the ratio of polymers presented in the continuous phase. For example, at low oil content (18%), the polymers needed to stabilize the oil droplets were less, and other non-adsorbed polymers could be used to form a continuous network by associated with one another. At high oil content (72%) (Figure 8F), most polymers were used to stabilize the oil–water interfaces to form smaller emulsion droplets, and less polymers were available for network formation. In this case, the empty spaces of polymers networks were enlarged, that meant more oil droplets were filled into the gel network. Similar to the study of Torquato et al. [37], who found when the oil content approached to 60%, the emulsions started to resemble a closed packed system.

### 2.8. Comparative Analysisof Sensory Characteristics of SFC and Commercial Cosmetic Moisturizers

**Figure 9:**
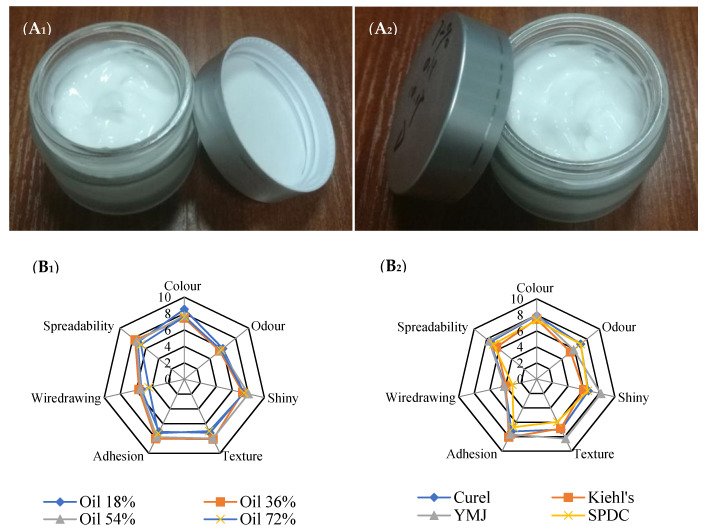

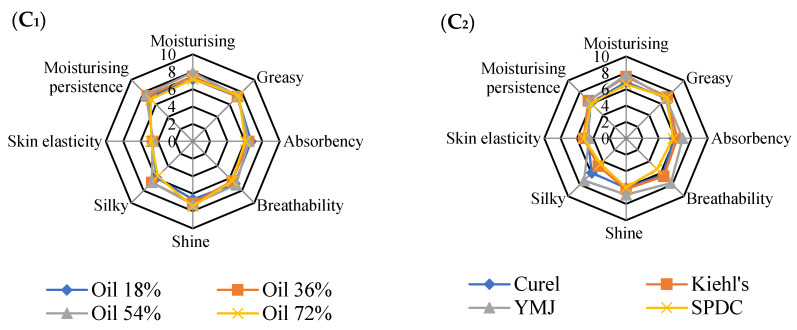
Quantitative results of the sensory evaluation of the investigated SFC (**A_1_**,**A_2_**,**B_1_**,**C_1_**) with various oil phase ratio and commercial cosmetic moisturizers (**B_2_**,**C_2_**).

As shown in Figure 9(A1,A2), the surface of SFCs was smooth, texture was uniform, and color was bright. In the sensory evaluation of color, shiny, texture, and adhesion, SFCs had advantages over the four commercial moisturizers but were slightly lacking in odor (Figure 9B). Among the samples, the SFC with oil content of 18% had the best color and odor, and that with oil content of 54% was the shiniest. The sensory evaluation of SFC (oil, 72%) was slightly less than that with other oil content, except for the low score of wiredrawing sensation of the sample, the score grade of other indexes was above 6, indicating that its appearance was still acceptable. The SFC with oil content of 54% showed the highest shine, fine texture, and soft properties because it had small internal particles, resulting in high glossiness. In terms of the sensory characteristics of adhesion and wiredrawing, these two indexes are generally proportional to each other, that is, the easier the adhesion is, the stronger the wiredrawing feeling. The score of adhesion characteristic of the SFC with 36% oil content and 54% oil content was 8; hence, the wiredrawing sensation was similar. Its wiredrawing score (6) was higher than that of commercial moisturizers (4) (Figure 9B). The texture of SFC with oil content of 72% was the thickest, which was consistent with the results of high viscosity and thixotropy. Its adhesion, wiredrawing, and spreading properties were lower than those of other three kinds of SFCs.

Figure 9C shows that during and after rubbing, the SFC with 54% oil content had the best moisturizing property, followed by the SFC with 36% oil content, and the worst was the SFC with 18% and 72% oil contents. In terms of greasy property, the greasy feeling is usually determined by the specific gravity of the oil phase in the emulsion. In general, the thicker the sample is, the stronger its greasy feeling will be. According to the sensory evaluation, the SFC with oil content of 72% had the highest greasy, followed by the SFC with oil content of 54%. In terms of absorbability, the SFC with oil content of 18% and 36% performed best, and the absorbability of the four SFCs were better than that of the other three commercial moisturizers except YMJ. In terms of shine, the SFC with oil content of 72% showed poor shine before rubbing but better shine after rubbing on the skin. In terms of silky character, the SFC with oil content of 36% and 54% showed good silkiness. In addition, the SFC with 54% oil content had the best performance in elasticity and moisturizing persistence after rubbing. In summary, the four kinds of SFCs were basically similar to commercial moisturizers in terms of appearance and sensory properties before rubbing and during rubbing phase. However, the four SFCs with different oil content had different sensory properties in the after-feel phase. In terms of moisturizing, silky, absorbency, and other aspects, the prepared SFCs were superior to other commercial moisturizers except for YMJ moisturizer. On the whole, the SFCs conformed to the commercial characteristics of cosmetic creams in terms of sensory properties.

## 3. Materials and Methods

### 3.1. Materials

AG (Mw 576 kDa) was obtained from Greenfresh Food Stuff Co., Ltd. (Fujian, China). Stearoyl chloride (SC/C18, 97%) was purchased from Aladdin Reagent (Shanghai) Co., Ltd. (Shanghai, China). Kiehl’s moisturizer (50 mL) was purchased from L’Oréal China (Shanghai, China). Pechoin (SPDC) moisturizer (50 g) was purchased from Shanghai Pechoin Daily Chemical Co., Ltd. (Shanghai, China). Curel moisturizer (40 g) was purchased from Kao (China) Holding Co., Ltd. (Shanghai, China). Yumeijing (YMJ) moisturizer (110 g) was purchased from Yumeijing Group Co., Ltd. (Tianjin, China). MUJI moisturizing lotion (200 mL) was purchased from Muji (Shanghai, China) Commercial Co., Ltd. (Shanghai, China). OSM moisturizing lotion (100 mL) was purchased from OSM Group Co., Ltd. (Zhejiang, China). CBM_940_ (CAS. No.9007-20-9) was obtained from Shanghai Macklin Biochemical Technology Co., Ltd. (Shanghai, China). It ssolution of 0.5% concentration has viscosity from 40,000 cps (minimum) to 60,000 cps (maximum). Caprylic triglyceride, isopropyl palmitate, squalane, and sodium hyaluronate (Mw 1800–2200 kDa) were acquired from Shanghai Macklin Biochemical Technology Co., Ltd. (Shanghai, China). All chemicals were used as received without further purification. 

### 3.2. Methods

#### 3.2.1. Synthesis of AS

AS was prepared according to the method of starch modifying with some modification [38]. Briefly, AG (20.0 g) was firstly suspended in 100 mL of pyridine and then different contents of stearoyl chloride were added dropwise. After 3 h of reaction with stirring at 80 °C, the mixture was washed with ethanol and deionized water, the AS was obtained after dried, pulverized, and sieved. The degree of substitution (DS) was measured by the method of Teramoto and Shibata [39].

#### 3.2.2. Characterization of AS

The FT-IR spectrum was recorded on a Nicolet iS50 FT-IR spectrometer (Thermo Fisher Nicolet, Waltham, MA, USA) at room temperature. The AS powder was blended with KBr powder and pressed into thin slices before being determined. A wavenumber from 4000 cm^−1^ to 500 cm^−1^ was used for scanning.

^13^C-NMR spectra was recorded on a Bruker 500 Avance spectrometer (Bruker, Etlingen, Germany), at 120 MHz, 90° pulse length, 0.865 acq. time and a relaxation delay of 1.5 s. AG and AS were dissolved in DMSO-d6 for NMR experiments.

TGA of AS powder was performed on a thermogravimetric analyzer Q500 (TA Instruments, Newcastle, DE, USA) between 30 °C and 800 °C at a heating rate of 5 °C/min in a N_2_ atmosphere.

#### 3.2.3. Surface Tension (ST) and Interface Tension (IT)

The ST and IT of the AS solution at different concentrations (2–12 g/L, *w*/*v*) were determined according to the Wilhelmy slide method by using an automatic surface tensiometer (BZY-1, Shanghai Sunny Hengping Scientific Instrument Co., Ltd, Shanghai, China). 

#### 3.2.4. Emulsifying Ability (EA) and Emulsifying Stability Index (ESI)

The emulsifying ability (EA) and emulsifying stability index (ESI) of the AS were determined according to the method of Ren and Lamsal [40]. Briefly, AS solution at different concentrations (2–12 g/L, *w*/*v*) was prepared by dispersing a certain amount of AS in deionized water and heating them until they were completely dissolved. O/W emulsion with oil phase volume fraction of 40% (*v*/*v*) was prepared by adding a certain amount of Arawana soybean salad oil (Yihai Kerry, Xiamen, China) into AS solution and then the mixture solution was homogenized by using a high-speed homogenizer (DLAB D-160, DLAB Scientific Co., Ltd., Beijing, China) at 24,000 rpm for 3 min at 75 °C. EA was determined by pipetting 100 µL of each emulsion into 10 mL of 0.1% sodium dodecyl sulfate solution. After emulsion formation, absorbance (EA) was measured at 500 nm. The ESI was calculated as: ESI = A_0_ × 1/[A_0_ − A_1_], where A_0_ and A_1_ are the absorbance values obtained at 0 and 1 h, respectively. High ESI values indicate high emulsion stability.

#### 3.2.5. Preparation of SFC

The SFC was prepared as follows. First, the oil phase components (caprylic triglyceride, 5.00–20.00% (*v*/*v*); isopropyl palmitate, 5.00–20.00% (*v*/*v*); squalene, 4.00–16.00% (*v*/*v*); dimethicone, 3.00–12.00% (*v*/*v*); and sheabutter, 1.00–4.00% (*v*/*v*)) were mixed and heated to 75 °C under mechanical stirring. The polymers (AS, 1.20–1.80 wt%; CBM940, 0.10–0.30 wt%) were pre-dispersed in propylene glycol (3%, *v*/*v*) and glycerin (4%, *v*/*v*) at room temperature and then dissolved in distilled water at 80 °C. Oil phase was then added to the dissolved polymer solution at 75 °C under mechanical stirring for 1 min, and sodium hydroxide was added to the emulsion to reach a final pH of 6.5. The emulsion was then homogenized using a high-speed homogenizer (DLAB D-160, Beijing, China) at 24,000 rpm for 1 min. After added the preservative and compensation for lost moisture, the emulsions were stirred for additional minutes at room temperature, bottled, and finally stored at 4 °C for further analysis.

#### 3.2.6. Rheological Experiments

All rheological tests were carried out using a controlled stress rheometer (DHR, TA Instrument, New Castle, DE, USA). Flow tests were carried out using a parallel plate PMMA geometry (40 mm diameter, 1000 µm gap) at 25 °C. The shear rate was ranging from 0.1 to 100 s^−1^, flow properties were obtained by recording shear stress and viscosity values.

The thixotropic properties were carried out using a parallel plate PMMA geometry (40 mm diameter, 1000 µm gap) at 25 °C. The upward curve was determined by a shear rate ramp ranging from 0.1 to 100 s^−1^. The downward curve was determined by a shear rate ramp ranging from100 to 0.1 s^−1^. For each formulation, three repeated measurements were done and mean values were reported.

For the oscillatory frequency sweep tests, the linear viscoelastic range was firstly determined by a strain sweep (0.1–100%) at a constant frequency of 1 Hz. Frequency sweep tests were then carried out at an angular frequency from 0.1% to 100%, and the constant strain was set as 1%.The storage modulus (G′), loss modulus (G″), and loss tangent (tanδ) were obtained during the oscillatory frequency sweep test. All measurements were carried out in triplicate and average values were reported.

Creep recovery tests were carried out using parallel plate PMMA geometry (40 mm diameter, 1000 µm gap) at 25 °C. The constant stress was set as 1%and maintained for a period of 1200 s, and then compliance was measured during 800 s after stress removed [41]. All measurements were carried out in triplicate and average values were reported.

The temperature ramp tests were carried out using a automatic mode. The exposed edge of samples was covered with a thin layer of dimethicone to avoid dehydration. During the heating process, the heating range is set as 20–90 °C, the heating rate is 5 °C/min, the strain is fixed at 1%, and the oscillation frequency is 1.0 rad/s. During the cooling process, the cooling range was set as 90–20 °C, the cooling rate was 5 °C/min, the strain was fixed as 1%, and the oscillation frequency was 1.0 rad/s. All measurements were carried out in triplicate and average values were reported.

#### 3.2.7. Morphology and Microstructure of SFC

The morphology of SFC was observed under a 400× optical microscope (Motic B1 220PL, Motic China Group Co., Ltd., Xiamen, China). For SEM (Hitachi S4800, Tokyo, Japan), freeze-drying (24 h) was applied to remove moisture through sublimation to preserve the actual microstructure of the SFC, and the oil phase was then removed by petroleumether. The dried product was placed on a double-sided adhesive and coated with a gold layer to avoid charging under the electron beam.

#### 3.2.8. Sensory Evaluation of the SFC

According to the literature of Gilbert et al. [42], quantitative description analysis (QDA) was used to test the sensory characteristics of SFC (Table 1).

## 4. Conclusions

The AS with emulsifying, thickening and gel properties was successfully synthesized. The AS-CBM_940_ polymer combinations showed strongly synergistic action. The SFC was prepared by exploiting the AS-CBM_940_ polymer combinations. Rheological characterization revealed that SFCs with different properties can be formed by manipulating the AS and CBM_940_ concentration and controlling the pH and oil content. The prepared SFCs have characteristics similar to those of commercial cosmetics. The results may be useful for designing surfactant-free cosmetics with rheological properties and for producing medicine products, such as ointment and wound dressings.

## Figures and Tables

**Table 1:** Sample description word and corresponding intensity digital table.

Sensory Characteristic		Definition	Directional teRm/Scoring Standards
Before rubbing	Color	Degree to which the product looks clear, ranging from yellow to white	Yellow–white/0–9
	Odor	Amount of any type of odor, like vegetable oil, lard, grease, or shortening, sweet or maple.	Strong–weak/0–9
	Shiny	Degree to which the product looks shiny, oily, glossy, or sheen.	Slightly–very/0–9
	Texture	impression of the thickness of the sample when it is rubbed between a thumb and a forefinger	Thin–thick/0–9
	Sticky	Amount of the sample that stays on forefinger after short contact (2 s) with the sample in a container	A little–a lot/0–9
	Wiredrawing	Impression of the distance that the product will wiredraw after picking-up	Slightly–very/0–9
During rubbing	Spreadability	Impression of the area that the product will cover when spread over the skin	A little–a lot/0–9
	Moisturizing	Degree to which the product feels moist	A little–a lot/0–9
	Greasy	Degree to which the product feels greasy	Slightly–very/0–9
	Absorbency	Impression of the rate of absorption of the product into the skin	Slowly–quickly/0–9
	Breathability	Degree to which the product leaves an oily or waxy film or coating on the skin after rubbing	A little–a lot/0–9
	Sheen	Degree to which the product looks shiny, glossy, is iridescent or glittery after rubbing	A little–a lot/0–9
	Silky	Degree to which the product feels smooth, silky, or soft on fingers after rubbing	Slightly–very/0–9
After rubbing	Skin elasticity	Degree to which skin feels firmand elastic after rubbing	Slightly–very/0–9
	Moisturizing persistence	Degree to which the product leaves a moisturizing feeling on the skin after rubbing	A little–a lot/0–9

## References

[1] Muda H., Aziz A., Taher Z., Aziz R., Hasham R. (2017). Cosmeceuticals and Natural Cosmetics.

[2] Costa R., Santos L. (2017). Delivery systems for cosmetics—From manufacturing to the skin of natural antioxidants. Powder Technol..

[3] Venkataramani D., Tsulaia A., Amin S. (2020). Fundamentals and applications of particle stabilized emulsions in cosmetic formulations. Adv. Colloid Interfac..

[4] Varvaresou A., Iakovou K. (2015). Biosurfactants in cosmetics and biopharmaceuticals. Lett. Appl. Microbiol..

[5] Martins D., Rocha C., Dourado F., Gama M. (2021). Bacterial cellulose-carboxymethyl cellulose (BC: CMC) dry formulation as stabilizer and texturizing agent for surfactant-free cosmetic formulations. Colloid. Surf. A.

[6] Dickinson E. (2018). Hydrocolloids acting as emulsifying agents-How do they do it?. Food Hydrocoll..

[7] Xiao Q., Chen G., Zhang Y.H., Weng H.F., Cai M.H., Xiao A.F. (2020). Evaluation of a novel self-emulsifiable dodecenyl succinylated agarose in microencapsulation of docosahexaenoic acid (DHA) through spray-chilling process. Int. J. Biol. Macromol..

[8] Kouhi M., Prabhakaran M.P., Ramakrishna S. (2020). Edible polymers: An insight into its application in food, biomedicine and cosmetics. Trends Food Sci. Tech..

[9] Jamshidian M., Savary G., Grisel M., Picard C. (2014). Stretching properties of xanthan and hydroxypropyl guar in aqueous solutions and in cosmetic emulsions. Carbohydr. Polym..

[10] Singh B., Kumar A. (2020). Graft and crosslinked polymerization of polysaccharide gum to form hydrogel wound dressings for drug delivery applications. Carbohydr. Res..

[11] Zhang X.F., Gu X.Q., Wang X.D., Wang H.M., Mao S.R. (2018). Tunable and sustained-release characteristics of venlafaxine hydrochloride from chitosan–carbomer matrix tablets based on in situ formed polyelectrolyte complex film coating. Asian J. Pharm. Sci..

[12] Zhang Y., Ng W., Hu J., Mussa S.S., Ge Y. (2018). Formulation and in vitro stability evaluation of ethosomal carbomer hydrogel for transdermal vaccine delivery. Colloid. Surf. B.

[13] Winkler H., Vorwerg W., Rihm R. (2014). Thermal and mechanical properties of fatty acid starch esters. Carbohydr. Polym..

[14] Vanmarcke A., Leroy L., Stoclet G., Crépy L.D., Lefebvre J.M. (2017). Influence of fatty chain length and starch composition on structure and properties of fully substituted fatty acid starch esters. Carbohydr. Polym..

[15] Garg S., Jana A.K. (2011). Characterization and evaluation of acylated starch with different acyl groups and degrees of substitution. Carbohydr. Polym..

[16] Xiao Q., Weng H.F., Chen G., Xiao A.F. (2019). Preparation and characterization of octenyl succinic anhydride modified agarose derivative. Food Chem..

[17] Zhu Y.Q., Chen X., McClements D.J., Zou L.Q., Liu W. (2018). pH-, ion- and temperature-dependent emulsion gels: Fabricated by addition of whey protein to gliadin-nanoparticle coated lipid droplets. Food Hydrocoll..

[18] Benchabane A., Bekkour K. (2008). Rheological properties of carboxymethyl cellulose (CMC) solutions. Colloid Polym. Sci..

[19] Singh V., Sethi R., Tiwari A. (2009). Structure elucidation and properties of a nonionic galactomannan derived from the Cassia pleurocarpa seeds. Int. J. Biol. Macromol..

[20] Mao Y.Y., McClements D.J. (2013). Modification of emulsion properties by hetero-aggregation of oppositely charged starch-coated and protein-coated fat droplets. Food Hydrocoll..

[21] Adeli M., Samavati V. (2015). Studies on the steady shear flow behavior and chemical properties of water-soluble polysaccharide from Ziziphus lotus fruit. Int. J. Biol. Macromol..

[22] Huang Y.B., Shi F.L., Wang L.M., Yang Y., Khan B.M., Cheong K.L. (2019). Preparation and evaluation of Bletillastriata polysaccharide/carboxymethyl chitosan/Carbomer940 hydrogel for wound healing. Int. J. Biol. Macromol..

[23] Wei Y.X., Lin Y.B., Xie R., Xu Y.F., Yao J. (2015). The flow behavior, thixotropy and dynamical viscoelasticity of fenugreek gum. J. Food Eng..

[24] Liu J.Z., Wang R.K., Gao F.Y., Zhou J.H., Cen K.F. (2012). Rheology and thixotropic properties of slurry fuel prepared using municipal waste water sludge and coal. Chem. Eng. Sci..

[25] Glicerina V., Balestra F., Rosa M.D., Romani S. (2013). Rheological, textural and calorimetric modifications of dark chocolate during process. J. Food Eng..

[26] Kurt A., Cengiz A., Kahyaoglu T. (2016). The effect of gum tragacanth on the rheological properties of salep based ice cream mix. Carbohyd. Polym..

[27] Patel A.R., Dumlu P., Vermeir L., Lewille B., Lesaffer A. (2015). Rheological characterization of gel-in-oil-in-gel type structured emulsions. Food Hydrocoll..

[28] Piau J.M. (2007). Carbopol gels: Elasto viscoplastic and slippery glasses made of individual swollen sponges: Meso- and macroscopic properties, constitutive equations and scaling laws. J. Non-Newton. Fluid Mech..

[29] Moller P., Fall A., Chikkadi V., Derks D., Bonn D. (2009). An attempt to categorize yield stress fluid behavior. Phil. Trans. Math. Phys. Eng. Sci..

[30] Manoi K., Rizvi S. (2009). Emulsification mechanisms and characterization of cold, gel-like emulsions produced from texturized whey protein concentrate. Food Hydrocoll..

[31] Fu L., Tang C.H. (2011). Cold, gel-like whey protein emulsions by microfluidisation emulsification: Rheological properties and microstructures. Food Chem..

[32] Line V.L.S., Remondetto G.E., Subirade M. (2005). Cold gelation of beta-lactoglobulinoil-in-water emulsions. Food Hydrocoll..

[33] De Vries A., Nikiforidis C.V., Scholten E. (2014). Natural amphiphilic proteins astri-block Janus particles: Self-sorting into thermo-responsive gels. Europhys. Lett..

[34] Dolz M., Hernández M.J., Delegido J. (2008). Creep and recovery experimental investigation of low oil content food emulsions. Food Hydrocoll..

[35] Yilmaz M.T., Karaman S., Dogan M., Yetim H., Kayacier A. (2012). Characterization of O/W model system meat emulsions using shear creep and creep recovery tests based on mechanical simulation models and their correlation with texture profile analysis (TPA) parameters. J. Food Eng..

[36] Rajinder P. (2006). Rheology of high internal phase ratio emulsions. Food Hydrocoll..

[37] Torquato S., Truskett T.M., Debenedetti P.G. (2000). Is random close packing of spheres well defined. Phys. Rev. Lett..

[38] Sun Y.J., Hu Q.G., Qian J.T., Li T., Ma P.M. (2016). Preparation and properties of thermoplastic poly(caprolactone) composites containing high amount of esterified starch without plasticizer. Carbohydr. Polym..

[39] Teramoto N., Shibata M. (2006). Synthesis and properties of pullulan acetate. Thermal properties, biodegradability, and a semi-clear gel formation in organic solvents. Carbohydr. Polym..

[40] Ren K., Lamsal B.P. (2017). Synthesis of some glucose-fatty acid esters by lipase from *Candida antarctica* and their emulsion functions. Food Chem..

[41] Lorenzo G., Zaritzky N., Califano A. (2013). Rheological analysis of emulsion-filled gels based on high acyl gellan gum. Food Hydrocoll..

[42] Gilbert L., Picard C., Savary G., Grisel M. (2013). Rheological and textural characterization of cosmetic emulsions containing natural and synthetic polymers: Relationships between both data. Colloid Surf. A.

